# A novel effector CfEC92 of *Colletotrichum fructicola* contributes to glomerella leaf spot virulence by suppressing plant defences at the early infection phase

**DOI:** 10.1111/mpp.12940

**Published:** 2020-04-22

**Authors:** Shengping Shang, Bo Wang, Song Zhang, Guangli Liu, Xiaofei Liang, Rong Zhang, Mark L. Gleason, Guangyu Sun

**Affiliations:** ^1^ State Key Laboratory of Crop Stress Biology in Arid Areas and College of Plant Protection Northwest A&F University Yangling China; ^2^ Department of Plant Pathology and Microbiology Iowa State University Ames Iowa State USA

**Keywords:** apple, *Colletotrichum*, effector, host immune response, pathogenicity

## Abstract

The ascomycete fungus *Colletotrichum fructicola* causes diseases on a broad range of plant species. On susceptible cultivars of apple, it induces severe early defoliation and fruit spots, named glomerella leaf spot (GLS), but the mechanisms of pathogenicity have remained elusive. Phytopathogens exhibit small secreted effectors to advance host infection by manipulating host immune reactions. We report the identification and characterization of CfEC92, an effector required for *C. fructicola* virulence. CfEC92 is a *Colletotrichum*‐specific small secreted protein that suppresses BAX‐triggered cell death in *Nicotiana benthamiana*. Accumulation of the gene transcript was barely detectable in conidia or vegetative hyphae, but was highly up‐regulated in appressoria formed during early apple leaf infection. Gene deletion mutants were not affected in vegetative growth, appressorium formation, or appressorium‐mediated cellophane penetration. However, the mutants were significantly reduced in virulence toward apple leaves and fruits. Microscopic examination indicated that infection by the deletion mutants elicited elevated deposition of papillae at the penetration sites, and formation of infection vesicles and primary hyphae was retarded. Signal peptide activity, subcellular localization, and cell death‐suppressive activity (without signal peptide) assays suggest that CfEC92 could be secreted and perform virulence functions inside plant cells. RNA sequencing and quantitative reverse transcription PCR results confirmed that the deletion mutants triggered elevated host defence reactions. Our results strongly support the interpretation that CfEC92 contributes to *C. fructicola* virulence as a plant immunity suppressor at the early infection phase.

## Graphical abstract

The secreted protein CfEC92 of *Colletotrichum fructicola* suppresses BAX‐triggered programmed cell death in *Nicotiana benthamiana* and enhances pathogenicity on apple leaves and fruits by interfering with host resistance.

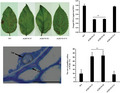

## 1 INTRODUCTION

In natural environments, plants are attacked by numerous pathogens and evolve sophisticated immunity surveillance systems to protect themselves (Jones and Takemoto, 2004; Chisholm *et al* *.*, 2006). A “zigzag” model summarizes the innate immunity in plant–pathogen interactions (Jones and Dangl, 2006). The pattern recognition receptors (PRRs) of plant cell surface receptors can detect pathogen‐associated molecular patterns (PAMPs) in the first line of defence, called PAMP‐triggered immunity (PTI). In turn, successful pathogens interfere with PTI by means of secreting effectors into host cells, resulting in effector‐triggered susceptibility (ETS). After that, plants activate the second layer of immune system through resistance (R) proteins that recognize pathogen effectors directly or indirectly, known as effector‐triggered immunity (ETI). However, successful pathogens can evolve new effectors that translocate into host cells to interfere with the plant immune system, facilitating pathogen colonization (Dou and Zhou, 2012; Dangl *et al* *.*, 2013; Wang and Wang, 2018).

*Colletotrichum* is a large genus of ascomycete plant pathogens infecting diverse field crops, vegetables, and fruits worldwide (Bailey and Jeger, 1992; Prusky *et al*., 2000). Most *Colletotrichum* species have a hemibiotrophic lifestyle. They develop penetration pegs from specialized melanized appressoria to invade living host cells, and then produce bulbous biotrophic infection vesicles and primary hyphae, before switching to a necrotrophic stage featuring secondary hyphae formation (Perfect *et al* *.*, 1999). Generally, phytopathogens deploy effectors to manipulate the host physiological metabolism and to suppress immune responses in order to establish biotrophy at an early phase of infection (Bozkurt *et al* *.*, 2012; Rafiqi *et al* *.*, 2012). Later, toxins and lytic enzymes are produced to kill and degrade host tissues in the necrotrophy mode (Gan *et al* *.*, 2012; O’Connell *et al* *.*, 2012).

Using genome sequencing and bioinformatics, a large number of effector candidate genes have been predicted genome‐wide in hemibiotrophic *Colletotrichum* species, including *C. gloeosporiodes*, *C. graminicola*, *C. higginsianum*,  and *C. orbiculare* (Crouch *et al* *.*, 2014). It has been suggested that each *Colletotrichum* species has evolved a unique set of effector genes to adapt to its own hosts (Crouch *et al* *.*, 2014). Furthermore, transcriptomic analysis of the infection process in *C. higginsianum* and *C. orbiculare* has indicated dynamic effector gene expression, supporting infection phase‐specific virulence roles of effector genes (Gan *et al* *.*, 2012; Kleemann *et al* *.*, 2012; O’Connell *et al* *.*, 2012). However, functional characterization of identified effector candidate genes has been lagging in *Colletotrichum*. So far, only a few effectors have been demonstrated to be important for virulence (Bhadauria *et al* *.*, 2012; Sanz‐Martin *et al* *.*, 2016), and in even fewer cases have the functional mechanisms been determined (Irieda *et al* *.*, 2014; Azmi *et al* *.*, 2018).

Glomerella leaf spot (GLS) is a disease of apple trees (*Malus domestica*) in Brazil, China, Japan, and the USA (Leite *et al* *.*, 1988; González and Sutton, 1999; Wang *et al* *.*, 2012; Yokosawa *et al* *.*, 2017). Under favourable conditions, GLS can result in more than 90% defoliation in susceptible cultivars such as Gala and Golden Delicious, seriously weakening the trees and reducing yield; fruits with spots are reduced in quality (Wang *et al* *.*, 2015b). In China, the disease occurs commonly from mid‐July to early August, a period of high temperature and humidity. In addition, the latent period is as short as 2 days and the disease spreads rapidly, especially after prolonged periods of rain. Therefore, control of GLS is challenging; to date, there are no consistently effective management approaches (Leite *et al* *.*, 1988; Wang *et al*., 2014).

Aetiological studies have identified *C. aenigma*, *C. alienum*, *C. fiorinae*, *C. fructicola*, *C. gloeosporioides*, *C. karstii*, *C. nymphaeae*, *C. siamense*, and *C. tropicale* as the GLS causal agents (Velho *et al* *.*, 2014, 2019; Wallhead *et al* *.*, 2014; Wang *et al* *.*, 2015a; Hoge, 2017), with *C. fructicola* the predominant one (Velho *et al* *.*, 2019). This species has a broad host range and its genome encodes the largest repertoire of candidate virulence genes, (e.g., small secreted proteins, cytochrome P450s, and plant cell wall‐degrading enzymes) among the GLS pathogens (Crouch *et al.*, 2014; Liang *et al.*, 2018). Transcriptomic comparison among infected apple leaves, conidia, in vitro appressoria, and infectious hyphae on cellophane has identified many candidate virulence genes showing in planta specific expression (Liang *et al.*, 2018). However, the functional importance of these candidate virulence genes has remained largely undetermined.

Based on the results of a transient expression assay in *Nicotiana*, we identified a novel effector, CfEC92, that inhibited BAX‐induced programmed cell death (PCD) and was highly expressed at the early infection phase. We also demonstrated that *CfEC92* gene deletion did not affect fungal vegetative growth or development, but strongly reduced virulence. This virulence reduction was related to elevated host defence reactions at the early infection phase and reduced efficiency in differentiation of infection vesicles and primary hyphae. Our results demonstrate that the CfEC92 is important for GLS virulence and provide insights regarding the molecular interactions between *C. fructicola* and its apple host.

## 2 RESULTS

### 2.1 CfEC92 is a *Colletotrichum* genus‐specific effector candidate with cell death‐suppressive activity

**FIGURE 1:**
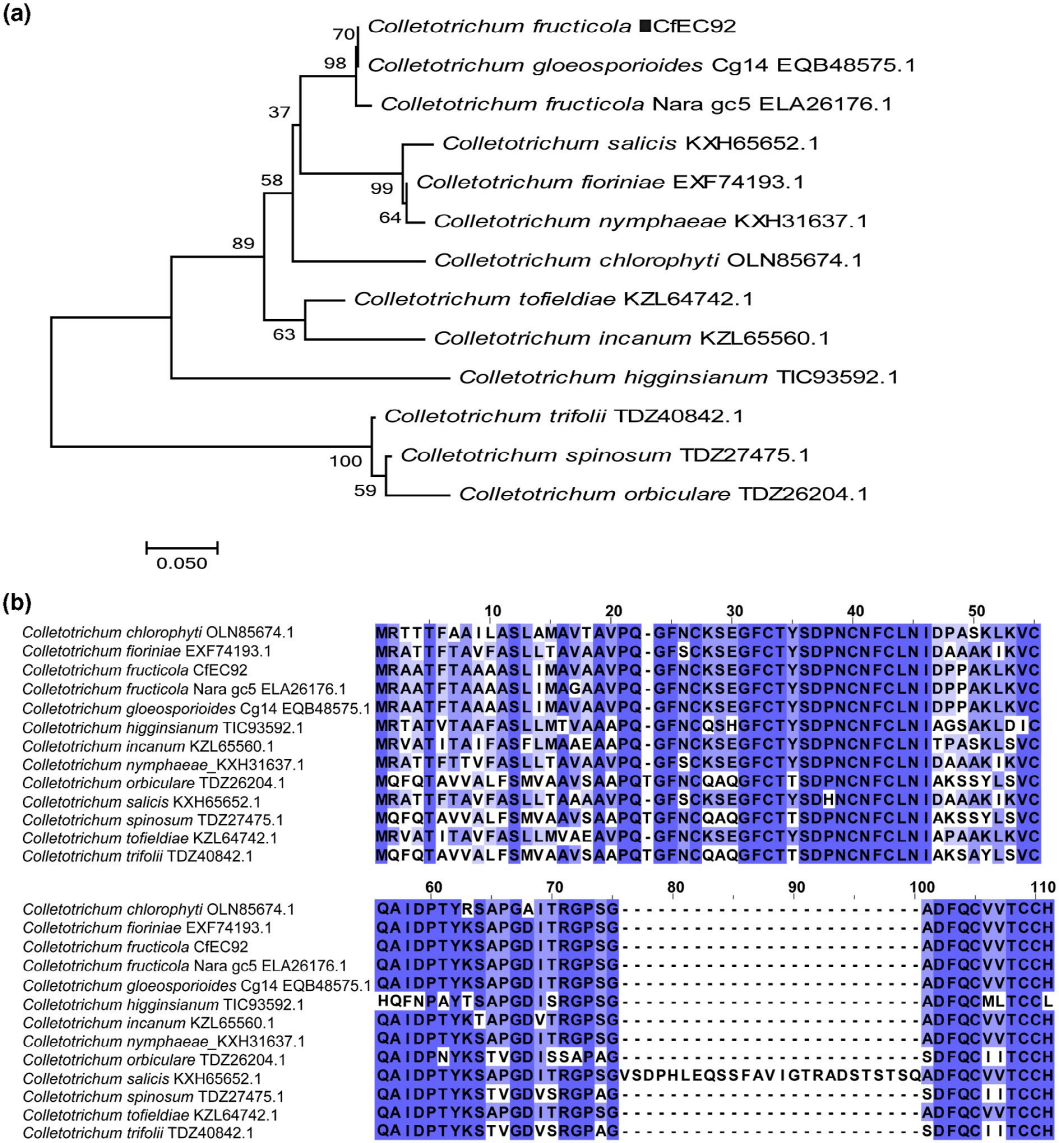
Sequence similarities between CfEC92 proteins among *Colletotrichum* species. (a) Phylogenetic analysis of CfEC92 and its homolog proteins in *Colletotrichum*. The full‐length protein sequences were analysed by MEGA 5 using unrooted neighbour‐joining bootstrap (1,000 replicates). The black box indicates CfEC92. The scale bar corresponds to a genetic distance of 0.05. (b) Multiple sequence alignment of CfEC92 and its homolog proteins. Full‐lengths of protein sequences were aligned using ClustalW and the alignment was edited using JalView. Blue shading intensity reflects the level of amino acid identity at each position

Among the effector candidates previously identified in *C. fructicola* (Liang *et al.*, 2018), *CfEC92* (1,104|04,129) showed in planta specific expression compared with conidia, in vitro appressoria, and infectious hyphae on cellophane. The gene encodes a putative protein with 85 amino acid residues and has a high cysteine content (9.41%). The protein was predicted to contain an N‐terminal signal peptide (SP) and showed 73.81% amino acid identity with ChEC34, a candidate effector gene of *C. higginsianum* whose expression is up‐regulated in appressoria (O’Connell *et al* *.*, 2012). A BlastP search against the National Center for Biotechnology Information (NCBI) non‐redundant (nr) database identified CfEC92 homologs in a range of additional *Colletotrichum* species (Figure 1), with amino acid identity ranging from 60.5% to  100%. However, no CfEC92 homologs were found in genera other than *Colletotrichum* (E‐value cut‐off = 1 × 10^−5^). We determined that CfEC92 is a lineage‐specific effector candidate that is conserved across the *Colletotrichum* genus.

**FIGURE 2:**
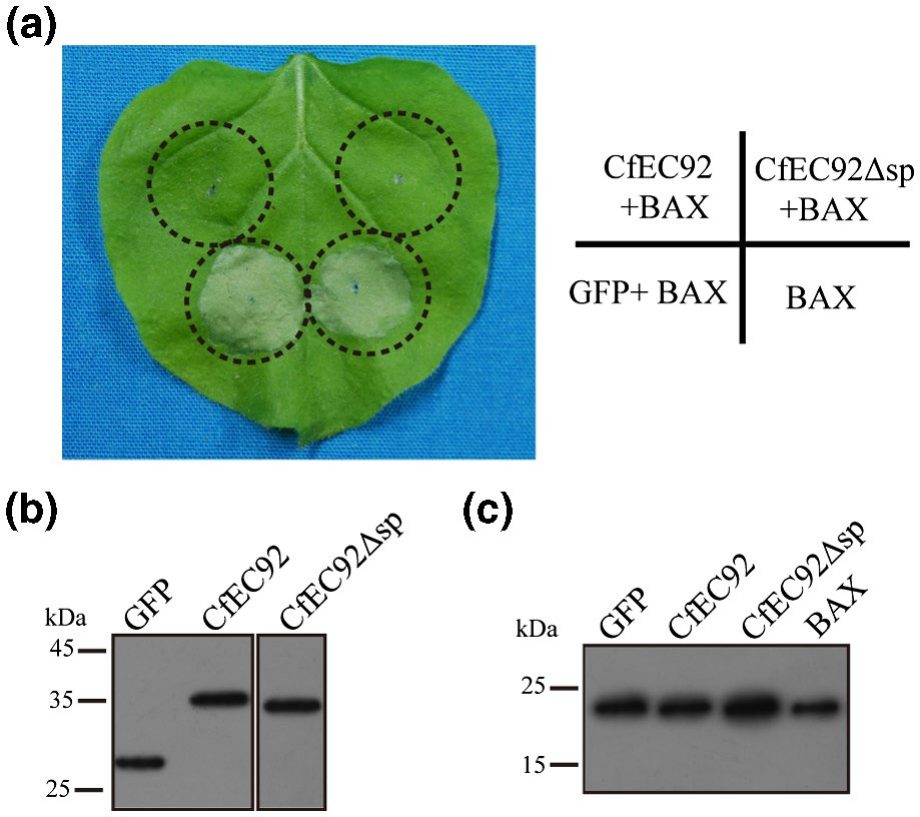
Transient expression of *CfEC92* in *Nicotiana benthamiana*. (a) CfEC92 with or without signal peptide (sp) suppressed BAX‐induced cell death. Leaves were infiltrated with *Agrobacterium tumefaciens* carrying the pGR107‐GFP vector inserted the *BAX* gene or the *CfEC92* gene with or without the signal peptide. Leaves were inoculated with empty pGR107‐GFP vector as a control. Red circles indicate the infiltrated region. Photographs were taken at 7 days after infiltration. The experiments were repeated nine times. (b) Western blot analysis of agroinfiltrated leaves expressing *CfEC92* with a green fluorescent protein (GFP) tag. The total proteins of infiltrating pGR107‐GFP vector were used as a control. (c) Western blot analysis of agroinfiltrated leaves expressing *BAX*

Transient overexpression of an effector candidate (EC) in *Nicotiana benthamiana* is a test method commonly used to investigate EC functions (Wang *et al* *.*, 2011). We have tested whether CfEC92 could suppress BAX‐induced cell death by co‐injecting *N. benthamiana* leaves with *Agrobacterium tumefaciens* overexpressing *CfEC92* and *A. tumefaciens* overexpressing *BAX*. As shown in Figure 2a, *BAX* gene expression strongly induced leaf necrosis at 7 days post‐inoculation. However, this cell death induction was totally suppressed by CfEC92 co‐infiltration. The same results were obtained in multiple independent infiltration assays. Western blot assays showed that co‐infiltration did not interfere with *BAX* gene expression (Figure 2b,c). These results strongly suggest that CfEC92 is a *Colletotrichum* genus‐specific effector with cell death‐suppressive activity.

### 2.2 CfEC92 transcript accumulation is highly up‐regulated at the early infection phase

**FIGURE 3:**
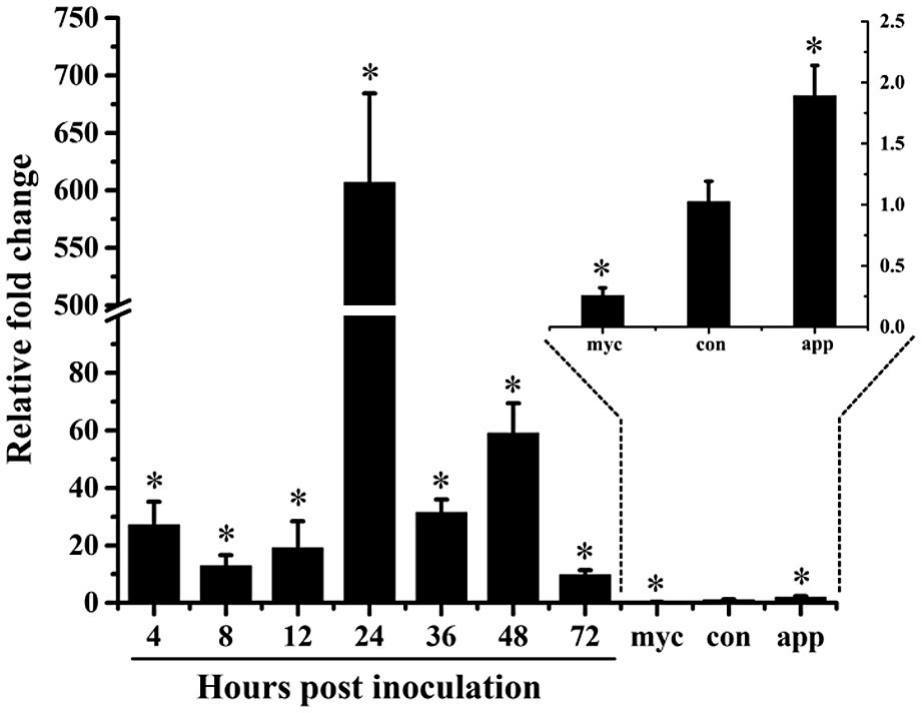
Quantitative reverse transcription PCR analysis of *CfEC92* expression patterns in conidia (con), mycelia (myc), appressoria (app) on cellophane, and inoculated leaves (4, 8, 12, 24, 36, 48, and 72 hr post‐inoculation) using *β‐tubulin* gene for normalization. Results are presented as the average fold‐change of three independent experiments compared to the conidia sample. Error bars represent *SE*. Asterisks indicate statistically significant differences (one‐way analysis of variance, *p* < .05)

Quantitative reverse transcription PCR (RT‐qPCR) was performed to compare the relative expression pattern of *CfEC92* among conidia, vegetative hyphae, and appressoria on apple leaves at different time points after inoculation. The transcript accumulation of *CfEC92* was barely detected in conidia, vegetative hyphae, and appressoria induced on cellophane (Figure 3). However, on inoculated apple leaves, *CfEC92* gene transcripts were induced significantly and reached a peak at 24 hr post‐inoculation (hpi) by around 600‐fold up‐regulation relative to conidia (Figure 3). During apple leaf infection, 24 hpi corresponded to appressorium formation and appressorium‐mediated penetration. We therefore concluded that *CfEC92* may play a role in virulence during appressorium formation at the early infection stage.

### 2.3 CfEC92 is specifically required for full expression of *C. fructicola* virulence

Two *CfEC92*‐deletion mutants (Δ*CfEC92*‐27 and Δ*CfEC92*‐52) were obtained by PCR screening of >20 hygromycin‐resistant transformants (Figure S1b). Reverse transcription (RT)‐PCR confirmed the lack of *CfEC92* transcript accumulation in these mutants during apple leaf infection (Figure S1c). The replacement of the *CfEC92* coding region by a hygromycin‐resistance gene was confirmed by RT‐PCR (Figure S1c) and Southern blotting (Figure S1d). Genetic complementation of the Δ*CfEC92*‐27 mutant was achieved by polyethylene glycol (PEG)‐mediated transformation and the obtained construct (Δ*CfEC92*‐27‐C) was also validated by RT‐PCR (Figure S1c).

**FIGURE 4:**
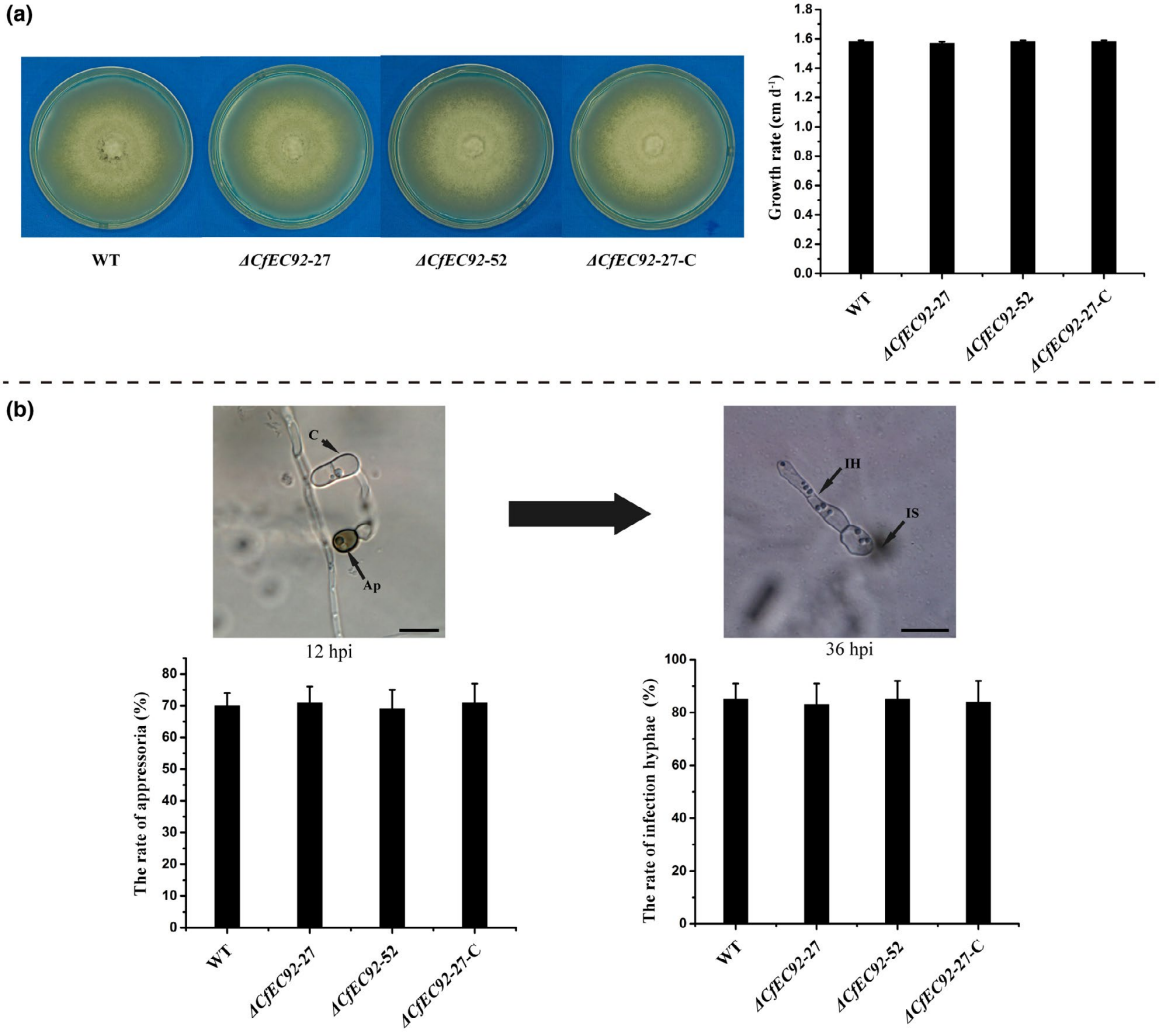
The effects of *CfEC92* deletion on the colony morphology, growth rate, appressoria formation, and infection hyphae formation of *Colletotrichum fructicola*. (a) Colony morphology and growth rate of wild type (WT), Δ*CfEC92*‐27, Δ*CfEC92*‐52, and Δ*CfEC92*‐27‐C (complemented) strains on potato dextrose agar for 7 days at 25 °C. (b) Appressoria and infection hyphae formation rate of WT, Δ*CfEC92*‐27, Δ*CfEC92*‐52, and Δ*CfEC92*‐27‐C strains on cellophane for 12 and 36 hpi at 25 °C, respectively. Experiments were performed three times. Values are presented as the means ± *SE*. C, conidia; Ap, appressoria; IH, infection hyphae. The red asterisk indicates the infection site. Bar: 10 μm

The knockout mutants showed no significant differences from the wild‐type strain 1104‐7 (WT) in vegetative growth on potato dextrose agar (PDA) (Figure 4a). In addition, the knockout mutants and WT were highly similar in appressorial development on cellophane (Figure 4b). By 36 hr after inoculation on cellophane, the frequency of infection hyphae from the knockout mutants was indistinguishable from WT and complementation strains (Figure 4b). These results demonstrated that *CfEC92* is not required for fungal vegetative growth, appressorium development, or appressorium‐mediated cellophane penetration.

**FIGURE 5:**
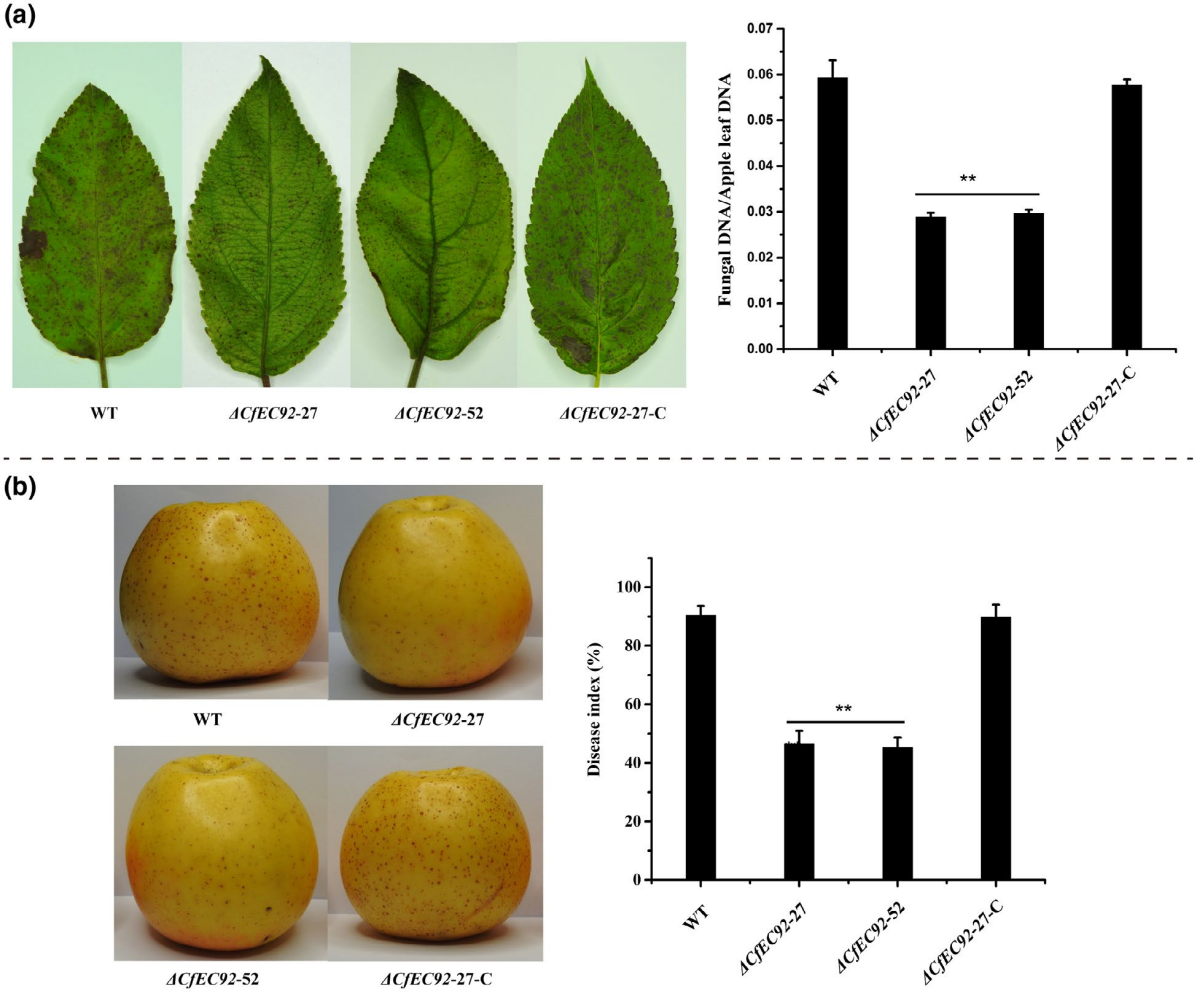
The deletion of *CfEC92* reduced the pathogenicity of *Colletotrichum fructicola* on apple leaves and fruits. (a) Δ*CfEC92* mutant strains reduced virulence on apple leaves compared to the wild type (WT) and complemented mutant strains. The virulence was estimated by fungal biomass relative to foliar biomass using quantitative PCR analysis of the *C. fructicola β‐tubulin* fragment relative to the apple *UBQ* fragment. The total DNA was extracted from apple leaves inoculated with WT, Δ*CfEC92*‐27, Δ*CfEC92*‐52, and Δ*CfEC92*‐27‐C (complemented) strains at 72 hr post‐inoculation, respectively. (b) Δ*CfEC92* mutant strains showed reduced virulence on apple fruits compared to wild‐type (WT) and complemented mutant strains. The virulence was evaluated by disease index. All experiments were performed for three replicates, each with nine leaves or fruits from three different apple trees. Values are presented as the means ± *SE*. The double asterisk indicates statistically significant differences (one‐way analysis of variance, *p* < .01)

We further tested the virulence of the knockout mutants and WT on apple leaves and fruit. After spraying the adaxial surface of detached apple leaves with a conidial suspension and incubating for 72 hr, the number of leaf spot lesions of the two mutants was significantly lower than that of the WT (Figure 5a). The relative in planta fungal biomass during mutant infection, as measured by quantitative PCR, was only half of that during WT infection. We also compared the virulence of different strains on apple fruit following conidial spray inoculation. Again, the *CfEC92* gene knockout mutant induced fewer necrotic lesions compared with the WT, and the disease index was reduced by approximately 50% at 5 days post‐inoculation (Figure 5b). Reintroduction of the *CfEC92* gene into the Δ*CfEC92*‐27 mutant strain resulted in restoration of virulence to the level of the WT in both leaf and fruit inoculation assays. These results strongly support the view that CfEC92 plays an infection‐specific role and is an important virulence factor in *C. fructicola*.

### 2.4 *CfEC92* suppresses plant defence reactions at the early infection phase

**FIGURE 6:**
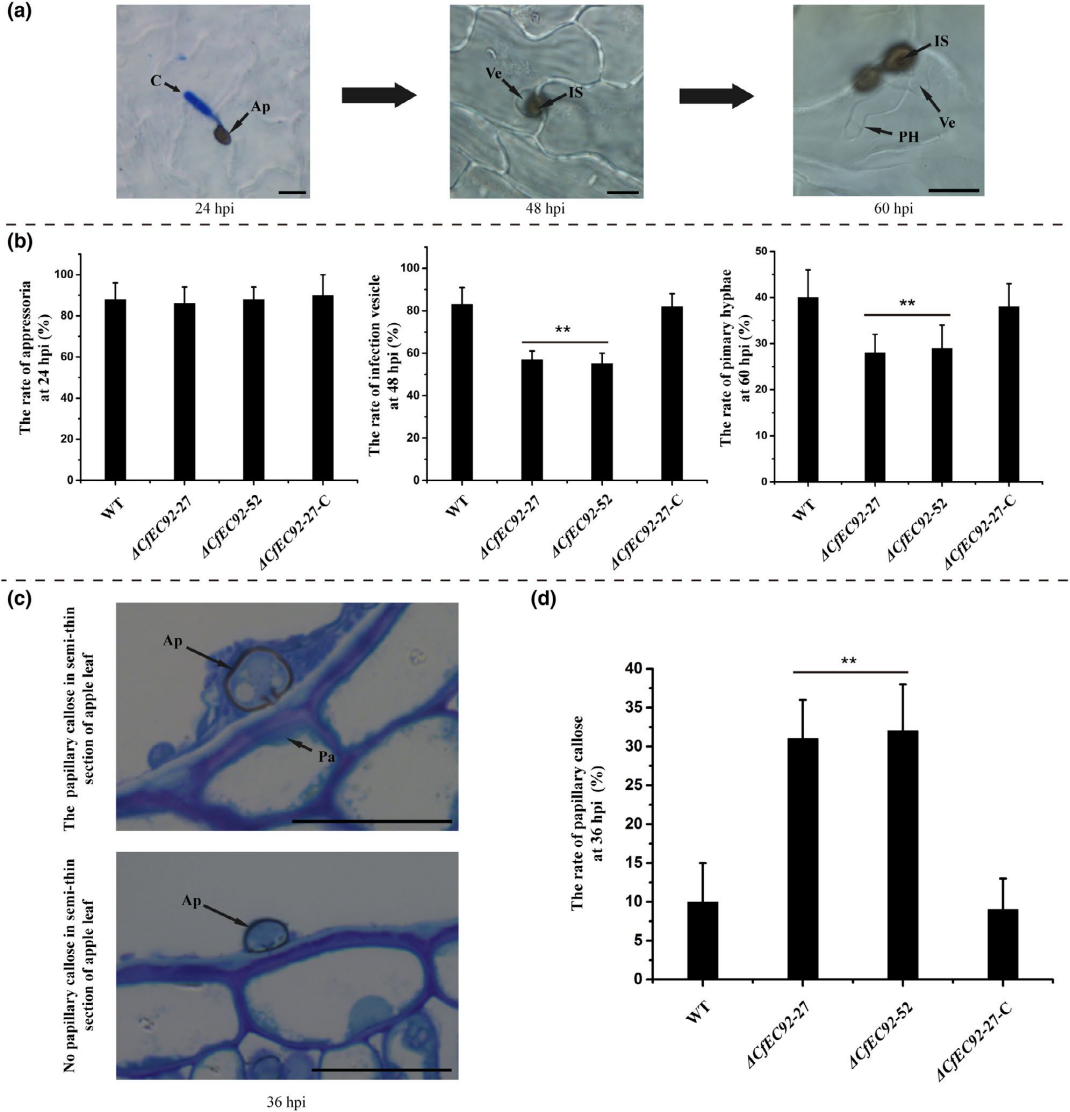
The deletion of *CfEC92* reduced the infection vesicle and primary hyphae formation, and increased papillary callose formation. (a) The different infection structures of *Colletotrichum fructicola* on apple leaves after 24, 48, and 60 hr post‐inoculation (hpi), respectively. (b) Formation rates of appressoria, infection vesicles, and primary hyphae of wild type (WT), Δ*CfEC92*‐27, Δ*CfEC92*‐52, and Δ*CfEC92*‐27‐C (complemented) strains on apple leaves at 25 °C after 24, 48, and 60 hpi, respectively. (c) Papillary callose of *C. fructicola* on apple leaves at 36 hpi. (d) Papillary callose formation of WT, Δ*CfEC92*‐27, Δ*CfEC92*‐52, and Δ*CfEC92*‐27‐C strains on apple leaves at 36 hpi at 25 °C. Experiments were performed three times. Values are presented as means ± *SE.* The double asterisk indicates statistically significant differences (one‐way analysis of variance, *p* < .01). C, conidia; Ap, appressorium; Ve, infection vesicle; Pa, papillary callose; PH, primary hyphae; IS, infection site. Bar: 10 μm

To determine the infection stage at which CfEC92 functions, we compared the histological infection events of different strains on apple leaves. At 24 hpi, frequency of appressorium formation was similar among the knockout mutant, WT, and complementation strains (Figure 6a,b). At 48 hpi, only 56% of appressoria of the knockout mutants could penetrate host epidermal cells and form visible infection vesicles, compared with 82% for WT (Figure 6a,b). We further quantified formation of primary hyphae at 72 hpi; formation rates of the mutants were 28%, approximately 12% lower than WT and complementation strains (Figure 6a,b). The reduced efficiency of infection vesicle formation in the Δ*CfEC92* mutant was correlated with increased papillae deposition in host epidermal cells at the penetration sites (Figure 6c,d). Frequency of papillae formation by the Δ*CfEC92* mutant was approximately 20% higher than that of WT and Δ*CfEC92*‐27‐C strains (Figure 6c,d). Taken together, it appears that deletion of *CfEC92* causes elevated host defence reactions at the penetration site, which in turn inhibits appressorium‐mediated penetration, infection vesicle development, and primary hyphae formation.

### 2.5 *CfEC92* exerts its virulence function inside plant cells

**FIGURE 7:**
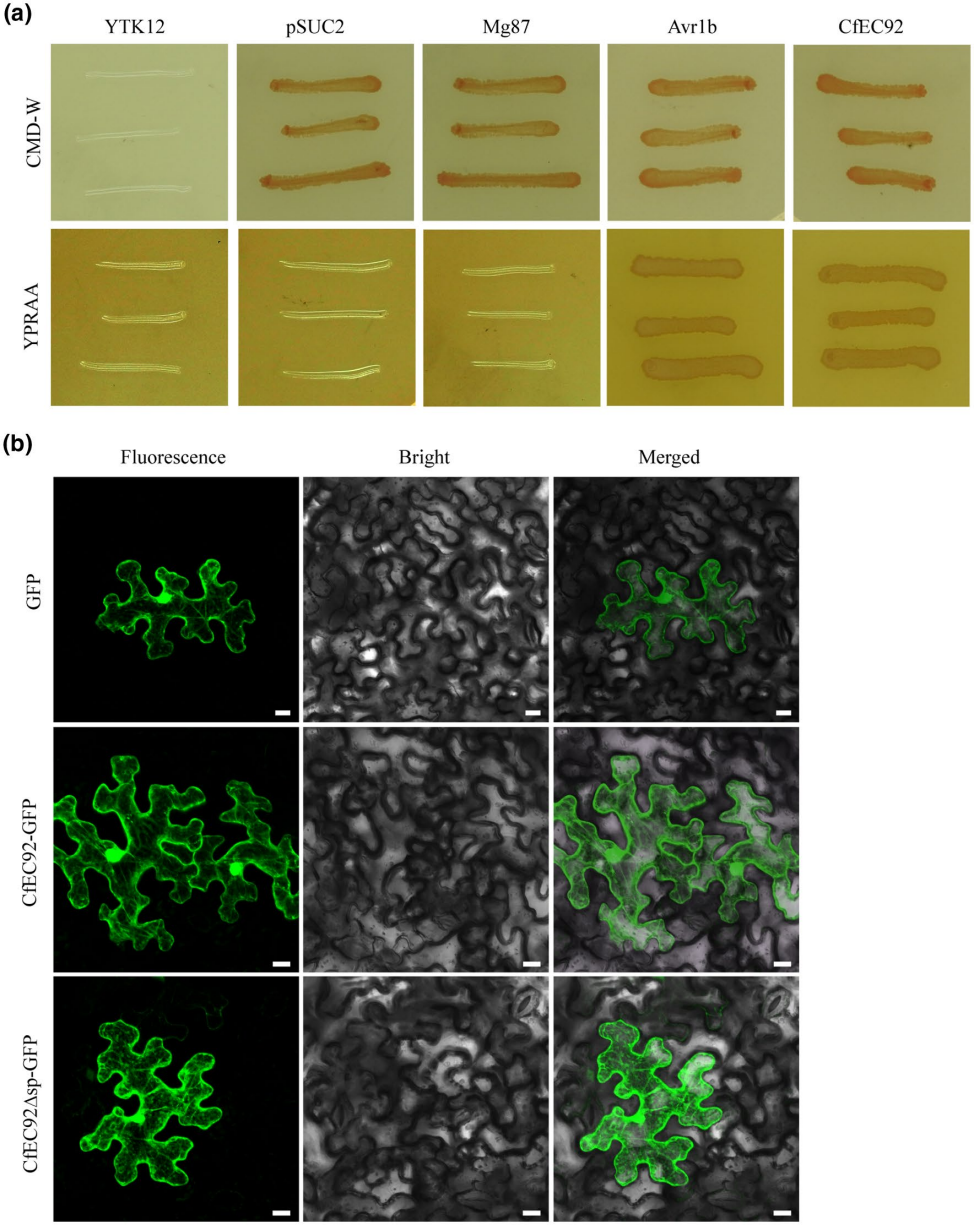
Functional validation of the putative signal peptide (SP) and localization of CfEC92. (a) The putative SP sequence of CfEC92 was fused in‐frame to the invertase gene in the pSUC2 vector and transformed into yeast strain YTK12. The YTK12 strains containing the empty pSUC2 and the first 25 amino acids of Mg87 protein from *Magnaporthe oryzae* were used as negative controls. The SP sequence of Avr1b from *Phytophthora sojae* was used as positive control. The transformed YTK12 strains that are able to secrete invertase were able to grow in both CMD‐W and YPRAA media. (b) *Agrobacterium tumefaciens* carrying green fluorescent protein (GFP), CfEC92‐GFP or CfEC92Δsp‐GFP fusion proteins was infiltrated into leaves of 5‐week‐old, *Nicotiana benthamiana*. Bar = 10 μm

SignalP 4.0 predicted the N‐terminal 19 amino acids of CfEC92 to be a putative SP. We validated the secretory function of this putative SP based on the yeast invertase secretion assay. When fused in‐frame to the yeast invertase sequence in the vector pSUC2, the predicted SP of CfEC92 mediated the complementation of the yeast YTK12 mutant strain (invertase deficient) in utilizing raffinose or growing on YPRA agar (Figure 7a). This result demonstrates that the predicted SP of CfEC92 is functional.

When fungal effectors are secreted, they function either in the apoplastic spaces or inside host cells after translocation. Because *CfEC92* overexpression suppressed BAX‐induced cell death, we tested whether this suppressive activity required a functional SP. In the tobacco transient expression assay, overexpression of CfEC92 lacking its N‐terminal SP still fully suppressed BAX‐induced cell death (Figure 2), supporting the interpretation that CfEC92 exerts its death‐suppressive function inside plant cells. Subcellular localization assay based on fusion with green fluorescent protein (GFP) also indicated that CfEC92 localizes in plant cells (Figure 7b).

### 2.6 Plant defence genes suppressed by CfEC92

**FIGURE 8:**
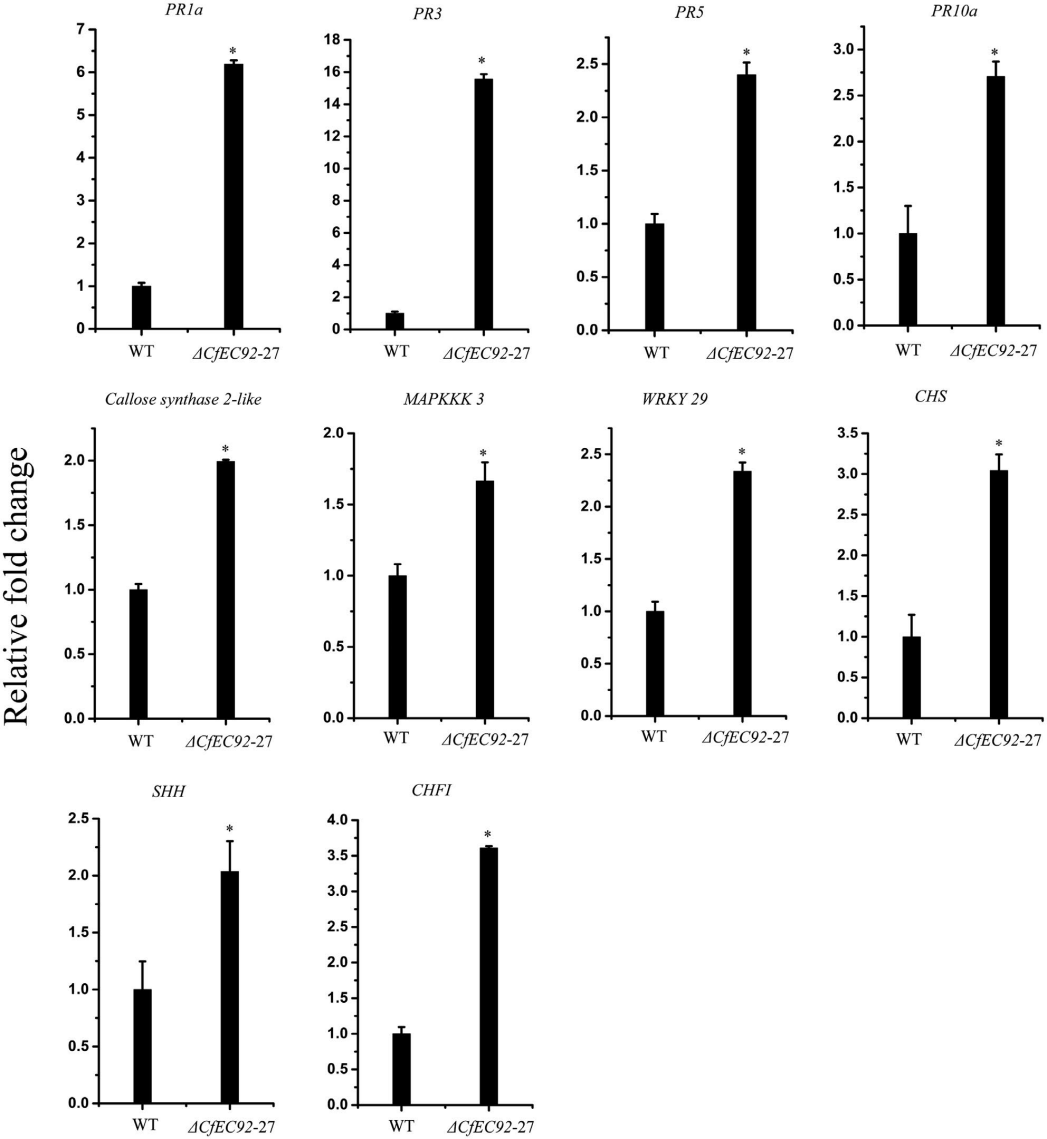
CfEC92 could suppress host immune responses. The total RNA was extracted from leaves inoculated with Δ*CfEC92*‐27 and wild‐type (WT) strains at 24 hr post‐inoculation and subjected to quantitative reverse transcription PCR analysis of 10 defence‐related genes. The vertical axis represents the relative fold changes of transcripts compared to the WT‐inoculated leaf sample and apple leaf *MdUBQ* was used as the reference gene. Error bars represent *SE*. Asterisks indicate statistically significant differences (one‐way analysis of variance, *p* < .05)

The increased deposition of papillae elicited by ∆*CfEC92* infection led us to perform an RNA sequencing (RNA‐Seq) experiment to compare the global expression patterns of host defence genes between WT and the ∆*CfEC92* mutant. Infected leaves were sampled at 24 hpi and subjected to RNA sequencing. A total of six libraries were constructed (three for WT and three for ∆*CfEC92*) and sequenced. In total, 52.41 Gb reads were obtained and mapped to the apple cv. Golden Delicious genome. A hierarchical clustering heatmap showed two distinct groups: one group containing the mutant strain and the other containing the WT strain, indicating that gene expression levels had low biological variability within the same treatment samples (Figure S2). We identified 310 differentially expressed genes that were expressed at least two‐fold higher in mutant infection relative to WT infection (Table S2). The up‐regulated genes included a range of plant defence‐related genes, such as pathogenesis‐related (*PR*) genes, *MAPKKK3*, *WRKY29*, and callose synthase. The up‐regulation of these defence‐related genes was further confirmed by RT‐qPCR (Figure 8). Pathway enrichment analysis revealed that six pathways, including 35 genes, were significantly (*t* test, *p* < .05) enriched, including glyoxylate and dicarboxylate metabolism (six genes), flavonoid biosynthesis (five genes), galactose metabolism (five genes), amino sugar and nucleotide sugar metabolism (seven genes), glycerolipid metabolism (five genes), and mitogen‐activated protein kinase (MAPK) signalling pathway (seven genes) (Figure S3).

Because flavonoids are important antifungal defence compounds (Falcone Ferreyra *et al* *.*, 2012), the enrichment of the flavonoid biosynthesis pathway implied the involvement of flavonoid compounds in defending against *C. fructicola* infection. The up‐regulated expression of five genes encoding three kinds of enzymes associated with flavonoid production implied that flavonoid synthesis increased to a greater extent in apple leaves inoculated with the Δ*CfEC92*‐27 strain than with the WT strain (Figure S4). In order to determine whether these flavonoids have a toxic role to *C. fructicola*, we measured the hyphal growth rate of *C. fructicola* after treatment by seven flavonoids on PDA. We found the tested flavonoids had different levels of antifungal properties against *C. fructicola* hyphae (Table 1 and Figure S5). The values of EC_50_ showed that 2′,3,4,4′,6′‐pentahydroxychalcone caused the greatest growth inhibition and 7,4′‐dihydroxyflavone was similar in inhibitory effect. These results suggests that CfEC92 is required to suppress the expression of a specific subset of defence‐related genes during the infection of *C. fructicola* on apple leaves.

**TABLE 1:** Toxicity of seven flavonoids on mycelial growth of *Colletotrichum  fructicola*

Flavonoid	Concentration (mg/L)	Regression equation of toxicity	Correlation coefficient (*r^2^*)	EC_50_ (mg/L)
2′,3,4,4′,6′‐Pentahydroxychalcone	1, 10, 20, 30	*y* = −0.0691*x* + 6.9287	0.92	36.6
7,4′‐Dihydroxyflavone	1, 10, 50, 70, 100	*y* = −0.014*x* + 6.5132	0.85	150.9
Liquiritigenin	1, 10, 50, 70, 100	*y* = −0.0111*x* + 6.2642	0.85	167.9
Naringenin	50, 100, 200, 300	*y* = −0.0192*x* + 7.1525	0.99	143.4
Phloretin	1, 10, 50, 70, 100	*y* = −0.0103*x* + 7.0159	0.92	254.0
Phlorizin	1, 10, 50, 70, 100	*y* = −0.0125*x* + 6.9148	0.95	194.9
Pinocembrin	1, 10, 50, 70, 100	*y* = −0.0124*x* + 7.3205	0.80	235.5

## 3 DISCUSSION

In most *Colletotrichum–*host interaction systems, *Colletotrichum* species exhibit a hemibiotrophic lifestyle. The early biotrophic infection stage is indispensable for completion of their life cycle. In this stage, these fungi often secret effectors to interfere with host defences and generate biotrophic infection structures for absorbing host cell nutrients (Gan *et al* *.*, 2012; O’Connell *et al* *.*, 2012). In addition, the genome of *C. fructicola* possess a large number of effector candidates and some of them show specifically up‐regulated expression during host infection (Liang *et al.*, 2018). Identification of effector candidates functioning at early infection stages will provide insights into the molecular mechanism of *C. fructicola–*apple leaf interaction.

Recognition of phytopathogenic PAMPs or effectors by host receptors would elicit strong host cell defence mechanisms such as the hypersensitive response (HR). The BAX protein, a member of the death‐promoting Bcl‐2 family, can trigger cell death when expressed in tobacco leaves using a virus vector (Lacomme and Cruz, 1999). The physiological process of induction of PCD in plants by the BAX protein is similar to the defence‐related HR (Lacomme and Santa Cruz, 1999). Therefore, it has been regarded as a valuable reference for pathogen effectors (Dou *et al* *.*, 2008). To date, many bacterial, oomycete, fungal, and nematode effectors have been identified using transient expression assays in the *N. benthamiana* model system employing agroinfiltration (Guo *et al* *.*, 2009; Wang *et al* *.*, 2011; Ali *et al* *.*, 2015). In the study, we demonstrated that CfEC92 suppressed BAX‐triggered cell death using a tobacco transient expression system, implying a defence‐suppressive function of CfEC92.

In the current study, the *Colletotrichum*‐genus specific effector CfEC92 was shown to be important for *C. fructicola* virulence, and its function in suppressing plant defence reactions was demonstrated. *CfEC92* was strongly induced in the early infection stage of the host plant, during progression from formation of appressoria to appearance of biotrophic infection vesicle. The knockout of *CfEC92* significantly reduced *C. fructicola* virulence in apple, indicating that CfEC92 contributed specifically to overcoming apple defences. In addition, secretion and subcellular assays suggested that CfEC92 was secreted from fungal cells and delivered into the host cells to influence the host. Further histological observations revealed that lack of CfEC92 significantly reduced the success rate of appressorium penetration due to the accumulation of papillary callose in host epidermal cells. Papillary callose is produced by callose synthase, and its deposition enhances the host cell's mechanical resistance to fungal appressorial pegs (Suzuki *et al*., 2003; Chen and Kim, 2009; Zhang *et al* *.*, 2011). In accordance with the histological results, the callose synthase gene was highly up‐regulated in the host inoculated with a *CfEC92* mutant compared to inoculation with the WT. Papillary callose deposition acts as a key factor for successful plant defence against microbial pathogens. Many plant pathogens, including bacteria, oomycete, and fungi, can secrete effectors to inhibit papillary callose formation (Tao *et al* *.*, 2003; Truman *et al* *.*, 2006; van den Burg *et al* *.*, 2006; Fabro *et al* *.*, 2011). For example, effector PSTha5a23 from *Puccinia striiformis* f. sp. *tritici* is involved in suppression of plant papillary callose formation (Cheng *et al* *.*, 2017). The core effector Cce1 contributes to early stages of infection of maize by *Ustilago maydis* via interfering with papillary callose deposition (Seitner *et al* *.*, 2018). Moreover, papillary callose deposition is an output of the PTI response. The transcriptome and RT‐qPCR experiments verified that the CfEC92 could suppress the expression of PTI marker genes, including *PR1a*, *WRKY29*, and *MAPKKK3* (Meng *et al* *.*, 2018; Sun *et al* *.*, 2018; Yang *et al* *.*, 2019). These findings suggest that CfEC92 is able to promote appressorium penetration through suppression of host PTI‐associated response.

Previous studies have reported that effector candidates are stage‐specifically expressed in the hemibiotrophic *Colletotrichum* stage switches, implying that the effectors may play an important role in the production of each infection structure of *Colletotrichum* species (Gan *et al* *.*, 2012; O’Connell *et al* *.*, 2012). In this study, we found an effector, CfEC92, that was associated with differentiation of primary hyphae from infection vesicles, and CfEC92 could inhibit host PTI response. These results suggest that host PTI‐related defence could restrain the formation of *C. fructicola* primary hyphae in apple leaf cells. It was also suggested that the formation of infection structures is the result of interaction between pathogen and host, which is not only related to pathogenesis genes (Dufresne *et al* *.*, 2000), but also to host resistance.

CfEC92 inhibited a subset of defence‐related gene expression. Comparative transcriptome analysis results showed that several defence genes involved in pathogenisis‐related (PR) proteins, the MAPK signalling pathway, and flavonoid synthesis were enriched in up‐regulated differential gene expression. The production and accumulation of PR proteins in plants are very important to resist phytopathogens (Ebrahim *et al* *.*, 2011). For example, PR3 can directly degrade chitin, a primary component of the fungal cell wall, to reduce the pathogenicity of an invading pathogen (Sharma *et al* *.*, 2011). In the study, the up‐regulated expressions of MAPKKK3 and WRKY29 were confirmed by RT‐qPCR. The two genes, as components of the MAPK cascades, are central to the transmission of biotic stress signals from membrane receptors into the nucleus, activating the downstream resistance responses to pathogens (Asai *et al* *.*, 2002; Pitzschke *et al* *.*, 2009). Flavonoids are associated with plant development and plant defence against pathogen and herbivores (Stafford, 1997; Shirley, 1998; Aoki *et al* *.*, 2000; Kliebenstein, 2004). For example, sakuranetin is a major phytoalexin in rice, effective against spore germination of *Pyricularia oryzae* (Kodama *et al* *.*, 1992). In this study, we found remarkably up‐regulated genes involving the biosynthesis of seven flavonoids that appeared to inhibit *C. fructicola* growth on PDA. Of these, naringenin has been reported to exert antimicrobial effects against *Staphylococcus epidermidis*, *Lactococcus lactis*, and *Candida krusei*, and phlorizin could directly inhibit growth of *Botrytis cinerea* in vitro (Nishino *et al* *.*, 1987; Uzel *et al*., 2005; Mandalari *et al* *.*, 2007; He *et al*., 2018); other flavonoids can exhibit pharmacological behaviour, such as anti‐inflammatory, antioxidant, and anticancer activity (Katavic *et al*., 2007; Mersereau *et al*., 2008; Rasul *et al*., 2013).

In summary, we identified a *Colletotrichum*‐specific effector (CfEC92) from *C. fructicola* suppressing BAX‐triggered PCD inside host cells. The CfEC92 functioned at an early stage of infection of *C. fructicola* on apple, helping to enable the pathogen to penetrate host cells from appressoria and facilitate differentiation of primary hyphae in apple leaves by suppressing host PTI and a subset of defence genes. However, the molecular mechanisms by which CfEC92 suppresses host defences and benefits the pathogenicity of *C. fructicola* remain unclear. Pathogenic effectors have been reported to target multiple host‐plant proteins, leading to suppression of host immunity responses (Kong *et al* *.*, 2017; Lan *et al* *.*, 2019). Because CfEC92 functions in host cells, our next work will focus on seeking the possible targets of CfEC92 in apple leaf cells in order to elucidate the molecular basis underlying plant defence suppression.

## 4 EXPERIMENTAL PROCEDURES

### 4.1 Fungal strains and plants culture conditions

The WT isolate of *C. fructicola*, 1104‐7, used in this study was maintained on PDA at 25 °C in the dark. For long‐term preservation, the isolate was stored at −80 °C. Knockout mutants of the *CfEC92* gene were maintained on PDA amended with 100 μg/ml hygromycin, whereas complementation strains were maintained on PDA amended with both hygromycin and G418 (Sangon Biotech). Seedlings of *N. benthamiana* were maintained in a greenhouse at 22–25 °C and 75% humidity with a light–dark cycle of 16/8 hr. For inoculation, fruit and healthy leaves of apple (*Malus domestica* 'Gala') were excised in our research orchard (34°17ʹ59″N, 108°03ʹ39″E) in Yangling, Shaanxi Province, China.

### 4.2 RNA extraction and RT‐qPCR

To clone and characterize the *CfEC92* expression during infection, the total RNA of conidia, mycelia, appressoria on cellophane, and inoculated *Malus domestica* 'Gala' leaves (4, 8, 12, 24, 36, 48, and 72 hpi) were extracted using an RNAprep Pure Plant Kit (TianGen Biotech) following the manufacturer's instructions. First‐strand complementary DNA (cDNA) was synthesized using the One‐Step gDNA Removal and cDNA Synthesis SuperMix (TransGen Biotech) following the recommended protocol.

RT‐qPCR was performed using an Applied Biosystems StepOnePlus Real‐Time PCR System with 2 × RealStar Green Power Mixture (GenStar) according to the manufacturer's operation manuals. Relative gene transcript levels were determined by the method of the comparative threshold cycle (*C*_t_) through StepOne v. 2.02 software installed in the real‐time PCR system. Normalized gene expressions were obtained by normalizing expression values to the conidia data using the *β‐tubulin* gene as an endogenous reference. Three independent technical replicates were performed per sample and the entire experiment (from leaf sample preparation to RT‐qPCR) was performed three times.

### 4.3 Plasmid construction

The *CfEC92* (accession number: MN604401) open reading frame (ORF) was amplified from cDNA (inoculated leaves) using *FastPfu* DNA polymerase (TransGen Biotech) and cloned into pEASY‐Blunt Zero vector (TransGen Biotech). In order to determine the SP secretion activity, the predicted SP sequence of *CfEC92* was fused in‐frame into the vector pSUC2 (Jacobs *et al.*, 1997). The SP of Avr1b and the N‐terminal 25 amino acids of Mg87 were joined with the vector pSUC2 as positive and negative controls, respectively (Shan *et al* *.*, 2004; Gu *et al* *.*, 2011). To determine the subcellular localization in *N. benthamiana*, the ORF sequence of *CfEC92* was cloned into the pBin‐eGFP vector. To perform the cell‐death inhibition assay in *N. benthamiana*, the coding sequence of *CfEC92* with or without the putative SP was fused with GFP sequence and then cloned into the pGR107 vector (Jones and Baulcombe, 1999). To complement the Δ*CfEC92* mutant, the entire *CfEC92* sequence containing the coding region and 1.5‐kb promoter was amplified and cloned into the vector pHZ‐100 containing G418 resistance gene. The primers used in this study are listed in Table S1.

### 4.4 Domain prediction, sequence alignment, and phylogenetic tree construction

The SP of CfEC92 was predicted using SignalP v. 4.1 (Nielsen, 2017). A search of CfEC92 homologs was performed using a BlastP search on the NCBI nr protein database. ClustalW was used for protein alignments and JalView was used for showing alignment results. An unrooted phylogenetic tree was constructed using MEGA 5 with the neighbour‐joining method (TreeBASE accession number 25276).

### 4.5 Cell‐death inhibition assay

The constructed vector pGR107 carrying *CfEC92* (with or without SP) was transformed into *A. tumefaciens* GV3101. For transient expression in *N. benthamiana*, recombinant strains of *A. tumefaciens* were cultured in Luria Bertani (LB) liquid medium containing kanamycin (50 µg/ml) and rifampicin (40 µg/ml) at 28 °C for 48 hr. Cells were resuspended in infiltration medium (10 mM MgCl_2_, 5 mM 2‐morpholinoethanesulfonic acid monohydrate [pH 5.7], and 150 µM acetosyringone) and adjusted to an OD_600_ = 0.6. Bacteria were infiltrated through a small incision with a needleless 1‐ml syringe into the lower leaves of 5‐week‐old *N. benthamiana* plants. Each of the recombinant *A. tumefaciens* strains was inoculated on three leaves on each of at least three plants. *A. tumefaciens* cells harbouring the *BAX* gene were infiltrated into the same sites on the leaves 24 hr later. The same leaves were infiltrated with *A. tumefaciens* carrying an empty vector pGR107 containing GFP as control. Cell death symptoms were observed and photographed at 7 days post‐infiltration. All tests were performed in triplicate.

### 4.6 Protein extraction and western blot

The total proteins of the leaf infiltrated area were extracted with a protein extraction kit (BB‐3124‐1, BestBio) in accordance with manufacturer's protocols. Western blotting was carried out to verify protein expression following standard procedures. Thirty microlitres of total protein per sample was loaded on 15% SDS‐polyacrylamide  gel for electrophoresis and then transferred to a polyvinylidene difluoride (PVDF) membrane (Millipore). For detecting effector and BAX proteins, mouse anti‐GFP monoclonal antibody (TransGen Biotech) and mouse anti‐BAX monoclonal antibody (Beyotime Biotechnology) were used as the primary antibodies, respectively. Goat anti‐mouse IgG‐peroxidase conjugate secondary antibody (TransGen Biotech) was used to detect the primary antibodies. The membrane was treated with an Easysee western blot kit (TransGen Biotech) for 1 min. Subsequently, the chemoluminescence signal of the PVDF membrane was exposed to BioMax (Kodak) light film and imaged using a ChemiDoc XRS system (Bio‐Rad).

### 4.7 CfEC92 gene knockout and complementation

The upstream and downstream flanking sequences of *CfEC92* were amplified. The resulting PCR products were fused with the hygromycin phosphotransferase (*hph*) fragments by double‐jointed PCR (Figure S1a). The joined upstream and downstream products were then transformed into protoplasts of *C. fructicola*. Transformants resistant to hygromycin were examined by PCR. Further deletion mutants were confirmed by Southern blot hybridization using the DIG DNA Labeling and Detection Kit II (Roche) according to the manufacturer's instructions. To complement the *CfEC92*‐deficient strain, the recombinant pHZ‐100‐*CfEC92* was introduced into protoplasts of Δ*CfEC92*‐27 mutant strain. Complementation strains were identified from G418‐resistant transformants using PCR.

### 4.8 Plant inoculation

To prepare a conidial suspension, potato dextrose broth (PDB) was inoculated with two 9‐mm diameter mycelial PDA plugs and shake cultured for 5 days at 28 °C and 180 rpm on a shaker (DHZ‐DA, Taichang Experimental Instrument). The culture medium was then filtered through a layer of Miracloth (Millipore) and centrifuged at 8,000 rpm for 3 min. Harvested conidia were washed three times, resuspended, and adjusted to a concentration of 5 × 10^6^ conidia/ml in sterile distilled water (SDW).

Leaves and fruit were sterilized by rubbing with 70% ethanol using cotton swabs, followed by wiping with sterile distilled water. After the water evaporated, the leaves and fruit were sprayed with the conidial suspension, then incubated for 48 hr in humid chambers (IKEA) at 25 °C in the dark.

Lesion diameters were measured after 6 days at 25 °C to compare the pathogenicity differences between WT, Δ*CfEC92*, and complemented mutant strains. To further assess differences in pathogenicity, we quantified the fungal biomass relative to apple leaf biomass among inoculated leaves at 72 hpi. Next, 50 ng genomic DNA was used to amplify the *C. fructicola β‐tubulin* fragment (*CfβTUB*) and DNA amounts were normalized to the *M. domestica* ubiquitin extension fragment (*MdUBQ*, MDU74358). At 5 dpi, the diameters of the fruit lesions were graded on the following scale: 1: ≤1 mm; 2: >1 mm, ≤2 mm; 3: >2 mm. Disease index (DI) was expressed as ∑(lesion number × associated lesion diameter)⁄(total lesion numbers × largest lesion diameter).

### 4.9 Microscopy

To evaluate the rate of appressoria formation, and infection vesicle and primary hyphae development of mutant and WT strains, five apple leaves inoculated by conidial suspension were sampled at 24, 48, and 60 hpi, respectively. At each time point, 20 leaf disks were obtained by a puncher, fixed for 3 days with ethanol/trichloromethane (3:1, vol/vol) containing 0.45% (wt/vol) trichloroacetic acid and then cleared in chloral hydrate (250 g/100 ml H_2_O) for 3 days. Leaf disks were stained in trypan blue for 10 min (Khan and Hsiang, 2003) and observed with 1,000× magnification under an X‐51 microscope (Olympus Corporation). Fifty spores were counted for each leaf disk. Simultaneously, their conidial suspensions were spread on cellophane appressed to the surface of water‐agar medium, and amounts of appressoria formation and infection hyphae were assessed at 12 and 36 hpi, respectively. For observation of the papillary callose of the host epidermal cell, inoculated leaves were sampled and treated as described by Arroyo *et al* *. *(2005). Semithin sections were cut to 0.5 μm thickness by an ultramicrotome (Ultra cut R; Leica), stained for 1 min with aniline blue, and examined using an X‐51 microscope.

### 4.10 SP activity assay

Constructed recombinant vectors were transformed into the yeast strain YTK12 as described by Gietz *et al. *(1995). All transformants were cultured on CMD–W medium (0.08% tryptophan dropout supplement, 0.65% yeast nitrogen base without amino acids, 2% sucrose, 0.1% glucose, and 2% agar) to select positive colonies. For testing invertase secretion, the successfully transformed yeast strains were cultured on YPRA agar (1% yeast extract, 2% peptone, 2% raffinose, 2 mg/ml antimycin A, and 2% agar).

### 4.11 Subcellular localization assay

To determine the subcellular localization of CfEC92, *A. tumefaciens* GV3101 containing the vector pBin‐eGFP with *CfEC92* was transiently expressed in *N. benthamiana* in an infiltration medium with an OD_600_ of 0.6. GV3101 carrying the empty pBin‐eGFP vector was used as control. At 2 days post‐infiltration, epidermal tissues of tobacco leaves were sampled and observed with an FV3000 laser scanning confocal microscope (Olympus Corporation) with a 488 nm emission filter.

### 4.12 Transcriptome analysis

To perform RNA‐Seq analysis, three independent biological samples of apple leaves inoculated with WT or Δ*CfEC92* mutant strain were collected for RNA extraction and sequencing. Paired‐end sequencing with a read length of 150 bp was performed by an Illumina HiSeq X10. Filtered reads were mapped to the genome of cv. Golden Delicious using TopHat v. 2.0.9 with the parameter “–g 1” (Trapnell *et al* *.*, 2009). The profile of gene expression levels was analysed using Cufflinks v. 2.2.1 (Trapnell *et al* *.*, 2010). Fragments per kilobase of transcript per million fragments mapped (FPKM) of transcripts that were greater than a two‐fold change (*p* < .05) were considered to be differentially expressed. Kyoto Encyclopedia of Genes and Genomes (KEGG) analysis was performed in an Omcishare cloud platform (http://www.omicshare.com). Genes of interest were selected for further confirmation by RT‐qPCR according to the procedure described above.

### 4.13 Fungal inhibition assays

Flavonoids were dissolved in 250 μl ethanol and diluted to 12.5 mL with PDA to different final concentrations (Table 1). A 9‐mm diameter mycelial plug of *C. fructicola* was transferred to each plate and incubated at 25 °C for 7 days. Two fungal diameters were measured perpendicular to each other; regression equations were used to calculate toxicity and EC_50_. A PDA plate containing 250 μl ethanol was used as a control treatment. All experiments were performed four times.

## Supporting information


**FIGURE S1** Construction and identification of Δ*CfEC92* and complementation mutants. (a) Schematic diagram showing the *CfEC92* gene replacement strategy. Small blue and red arrows indicate primer binding sites. (b) Validation of wild‐type (WT), Δ*CfEC92*, and complementation mutant strains by PCR analysis: (1) partial *hph* gene was amplified with primers HY/YG (lanes 1, 5, 9, 13); (2) upstream region of *CfEC92* was amplified with primers LF/Xu855R (lanes 2, 6, 10, 14); (3) downstream region of *CfEC92* was amplified with primers Xu866F/RR (lanes 3, 7, 11, 15); (4) partial region of targeted gene *CfEC92* was amplified with primers 92DF/92DR (lanes 4, 8, 12, 16). Lane M, DL5000 marker. (c) The expressions of *CfEC92* gene in WT, Δ*CfEC92*‐27, Δ*CfEC92*‐52, and Δ*CfEC92*‐27‐C strains were detected by RT‐PCR analysis with primers 92DF/92DR and the *β‐tubulin* gene served as the positive reference gene. Lane marker, DL5000 marker. (d) Southern blot hybridization analysis of WT and Δ*CfEC92* mutant strains. The *CfEC92* gene was detected using probe A amplified with primers T92F/T92R and the probe B of* hph* gene was amplified with primers THF/THR

**FIGURE S2** The hierarchical clustering heatmap of the nine samples used in this study shows two distinct clades: one consisted of the mutant strain and the other the WT strain

**FIGURE S3** The bubble diagram shows the significant (*p* < .05) pathway enrichment of differentially expressed genes in apple leaves inoculated with Δ*CfEC92*‐27 strain. The rich factor represents the ratio of differential genes to all annotated genes in the same pathway

**FIGURE S4** The metabolic pathways of some flavonoids, with the red boxes representing up‐regulated genes and the blue boxes representing compounds tested for inhibition of *Colletotrichum fructicola*. CHS, chalcone synthase; CHFI, chalcone‐flavonone isomerase 3; SHH, shikimate o‐hydroxycinnamoyltransferase‐like

**FIGURE S5** In vitro antifungal activity of seven flavonoids on *Colletotrichum fructicola*. The concentration gradients of flavonoid compounds appeared to have different inhibition effects on the growth of *C. fructicola *on potato dextrose agar. Control CK was treated with an equal volume of alcohol used for dissolving flavonoids. Photographs were taken at 6 or 7 days after treatment

**TABLE S1** Primers used in this study

**TABLE S2** Genes up‐regulated in apple leaves inoculated with a *CfEC92* knockout mutant

## Data Availability

The data that support the findings of this study are available from the corresponding author on reasonable request.
