# Supplementary figures and images for: A novel effector CfEC92 of Colletotrichum fructicola contributes to glomerella leaf spot virulence by suppressing plant defences at the early infection phase
Source: Mol Plant Pathol. 2020 Apr 22;21(7):936–50. doi: 10.1111/mpp.12940 (PMC7279981; doi:10.1111/mpp.12940)

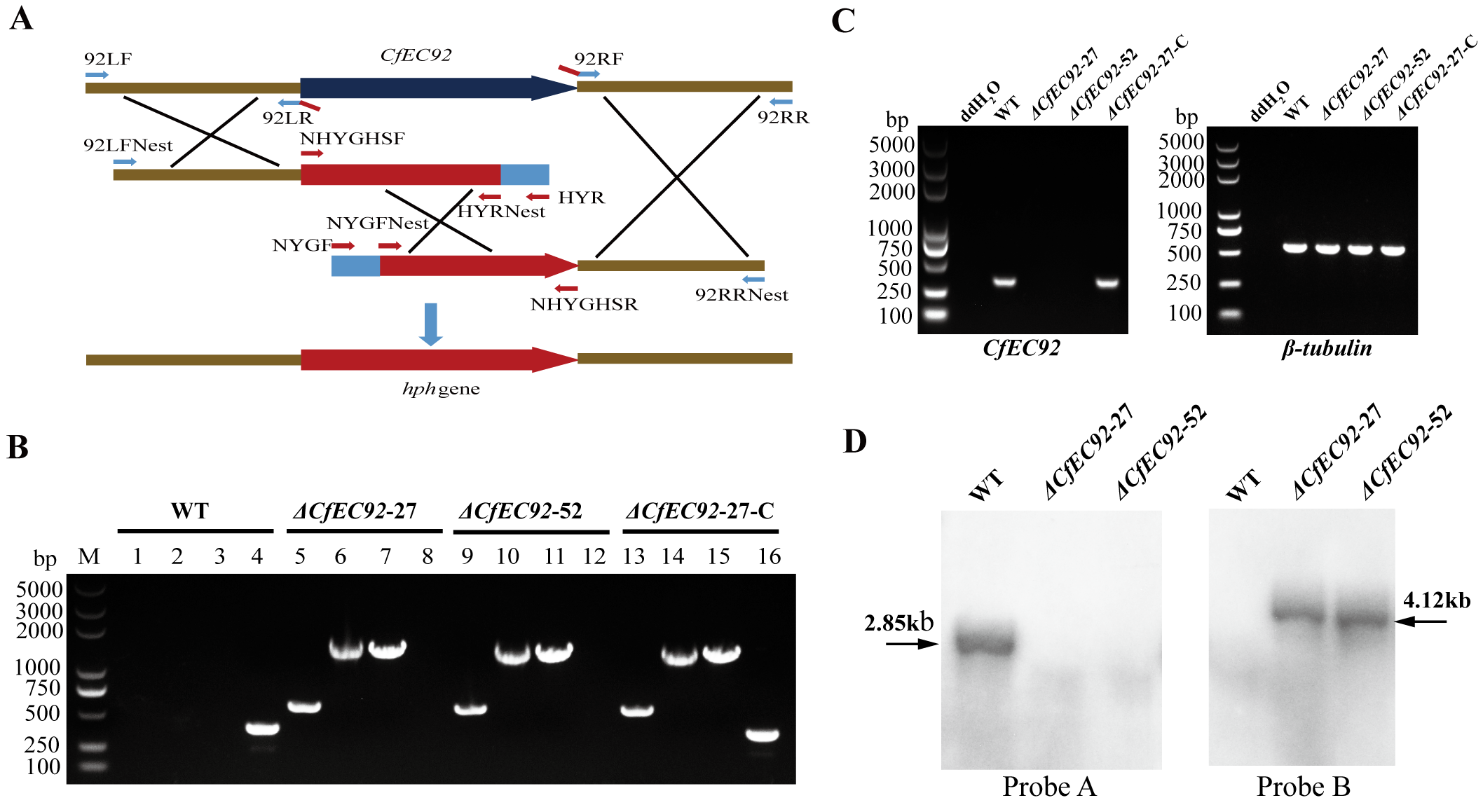

Supplement: Supplementary file 1 — FIGURE S1 Construction and identification of ΔCfEC92 and complementation mutants. (a) Schematic diagram showing the CfEC92 gene replacement strategy. Small blue and red arrows indicate primer binding sites. (b) Validation of wild‐type (WT), ΔCfEC92, and complementation mutant strains by PCR analysis: (1) partial hph gene was amplified with primers HY/YG (lanes 1, 5, 9, 13); (2) upstream region of CfEC92 was amplified with primers LF/Xu855R (lanes 2, 6, 10, 14); (3) downstream region of CfEC92 was amplified with primers Xu866F/RR (lanes 3, 7, 11, 15); (4) partial region of targeted gene CfEC92 was amplified with primers 92DF/92DR (lanes 4, 8, 12, 16). Lane M, DL5000 marker. (c) The expressions of CfEC92 gene in WT, ΔCfEC92‐27, ΔCfEC92‐52, and ΔCfEC92‐27‐C strains were detected by RT‐PCR analysis with primers 92DF/92DR and the β‐tubulin gene served as the positive reference gene. Lane marker, DL5000 marker. (d) Southern blot hybridization analysis of WT and ΔCfEC92 mutant strains. The CfEC92 gene was detected using probe A amplified with primers T92F/T92R and the probe B of hph gene was amplified with primers THF/THR [file MPP-21-936-s001.tif]

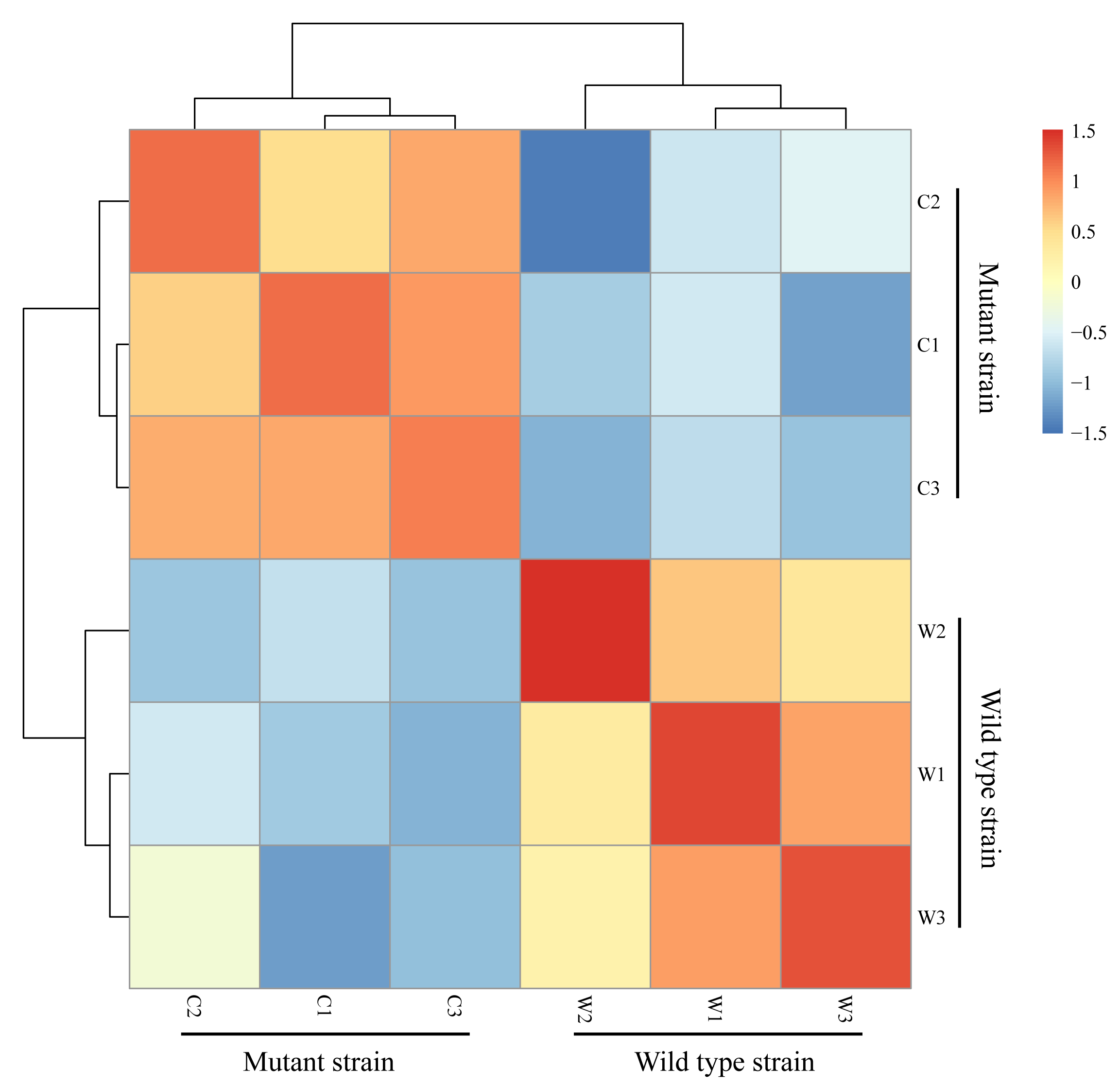

Supplement: Supplementary file 2 — FIGURE S2 The hierarchical clustering heatmap of the nine samples used in this study shows two distinct clades: one consisted of the mutant strain and the other the WT strain [file MPP-21-936-s002.tif]

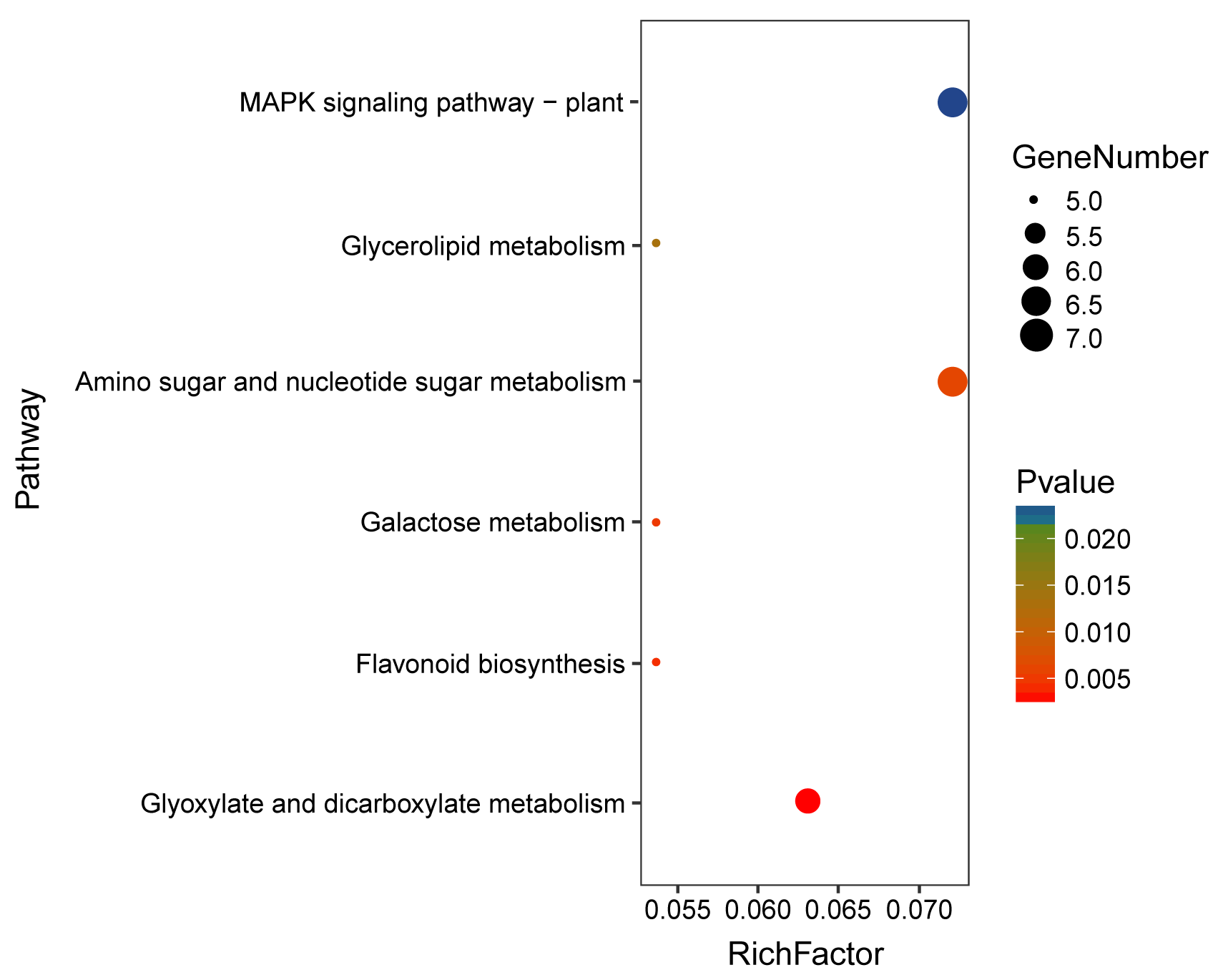

Supplement: Supplementary file 3 — FIGURE S3 The bubble diagram shows the significant (p < .05) pathway enrichment of differentially expressed genes in apple leaves inoculated with ΔCfEC92‐27 strain. The rich factor represents the ratio of differential genes to all annotated genes in the same pathway [file MPP-21-936-s003.tif]

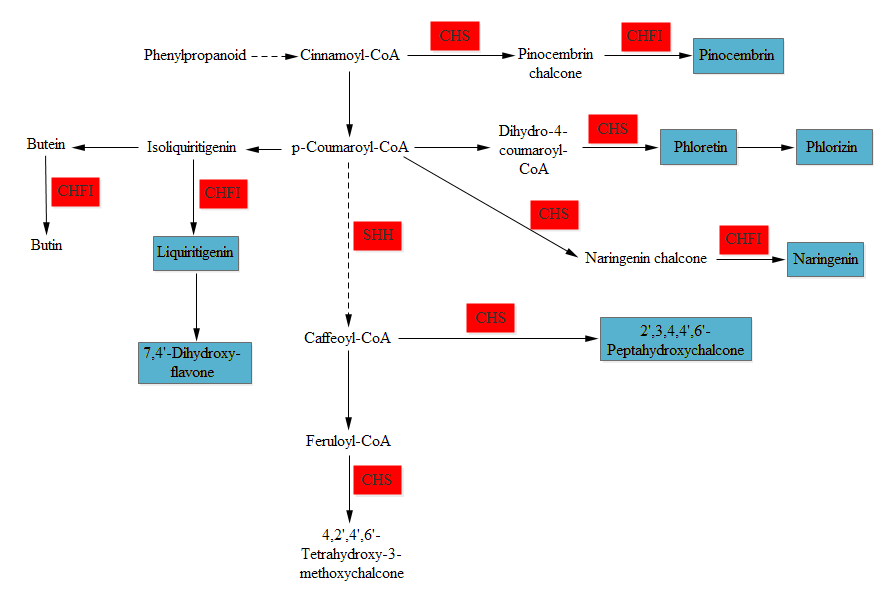

Supplement: Supplementary file 4 — FIGURE S4 The metabolic pathways of some flavonoids, with the red boxes representing up‐regulated genes and the blue boxes representing compounds tested for inhibition of Colletotrichum fructicola. CHS, chalcone synthase; CHFI, chalcone‐flavonone isomerase 3; SHH, shikimate o‐hydroxycinnamoyltransferase‐like [file MPP-21-936-s004.tif]

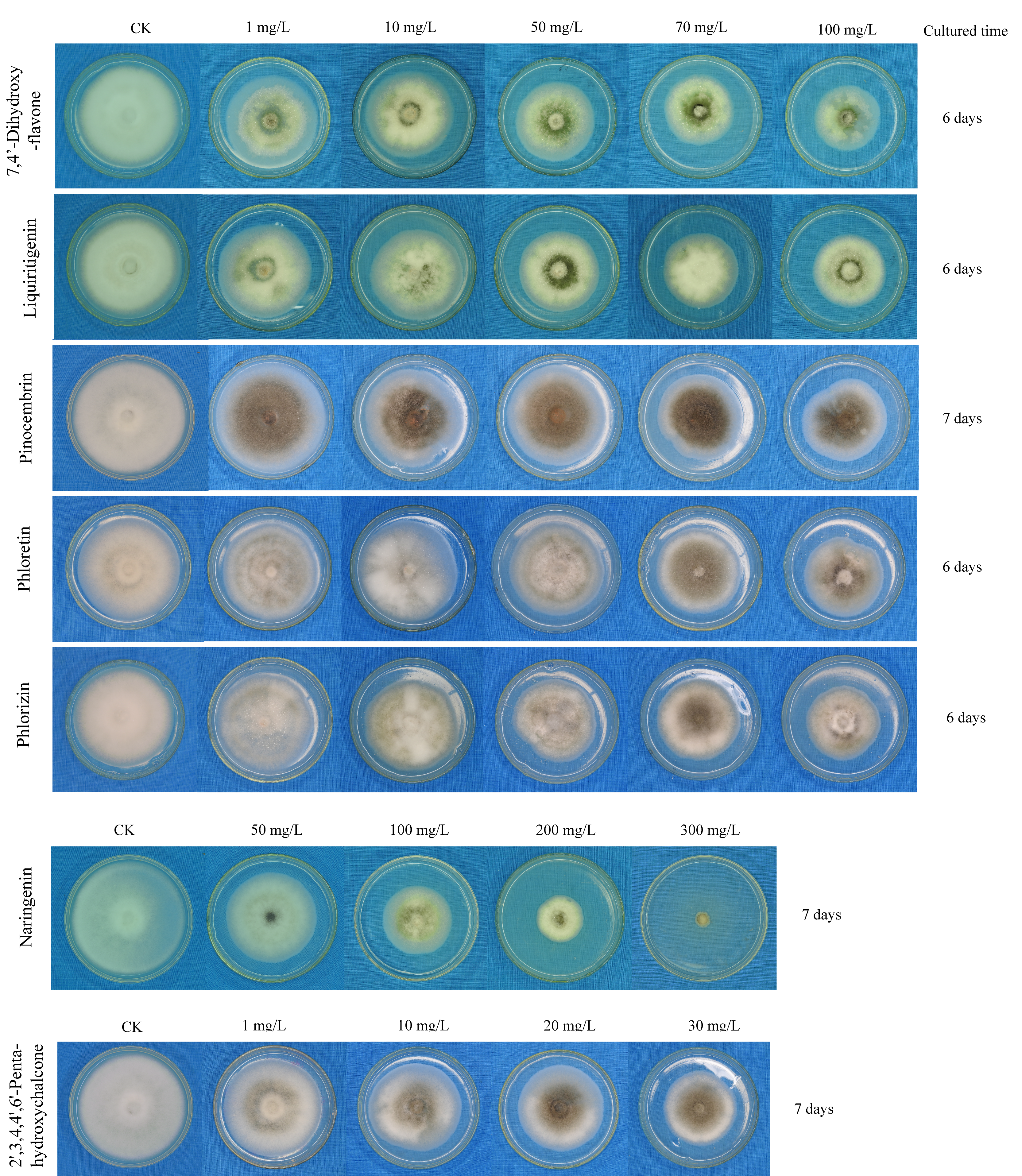

Supplement: Supplementary file 5 — FIGURE S5 In vitro antifungal activity of seven flavonoids on Colletotrichum fructicola. The concentration gradients of flavonoid compounds appeared to have different inhibition effects on the growth of C. fructicola on potato dextrose agar. Control CK was treated with an equal volume of alcohol used for dissolving flavonoids. Photographs were taken at 6 or 7 days after treatment [file MPP-21-936-s005.tif]
